# Effects of an invasive plant transcend ecosystem boundaries through a dragonfly-mediated trophic pathway

**DOI:** 10.1007/s00442-012-2357-1

**Published:** 2012-05-24

**Authors:** Laura A. Burkle, Joseph R. Mihaljevic, Kevin G. Smith

**Affiliations:** 1 Tyson Research Center, Washington University in St. Louis, Eureka, MO 63025 USA; 2 Present Address: Department of Ecology, Montana State University, Bozeman, MT 59717 USA; 3 Present Address: Department of Ecology and Evolutionary Biology, University of Colorado, Boulder, CO 80309 USA

**Keywords:** Bottom-up, Consumer–resource, Purple loosestrife, Top-down, Trophic interactions

## Abstract

Trophic interactions can strongly influence the structure and function of terrestrial and aquatic communities through top-down and bottom-up processes. Species with life stages in both terrestrial and aquatic systems may be particularly likely to link the effects of trophic interactions across ecosystem boundaries. Using experimental wetlands planted with purple loosestrife (*Lythrum salicaria*), we tested the degree to which the bottom-up effects of floral density of this invasive plant could trigger a chain of interactions, changing the behavior of terrestrial flying insect prey and predators and ultimately cascading through top-down interactions to alter lower trophic levels in the aquatic community. The results of our experiment support the linkage of terrestrial and aquatic food webs through this hypothesized pathway, with high loosestrife floral density treatments attracting high levels of visiting insect pollinators and predatory adult dragonflies. High floral densities were also associated with increased adult dragonfly oviposition and subsequently high larval dragonfly abundance in the aquatic community. Finally, high-flower treatments were coupled with changes in zooplankton species richness and shifts in the composition of zooplankton communities. Through changes in animal behavior and trophic interactions in terrestrial and aquatic systems, this work illustrates the broad and potentially cryptic effects of invasive species, and provides additional compelling motivation for ecologists to conduct investigations that cross traditional ecosystem boundaries.

**Electronic supplementary material:**

The online version of this article (doi:10.1007/s00442-012-2357-1) contains supplementary material, which is available to authorized users.

## Introduction

Consumer–resource interactions can strongly influence community structure and function via both top-down (predator-controlled) and bottom-up (producer-controlled) interactions (e.g., Fretwell 1987; Schmitz 2010). Predator- and producer-driven trophic patterns have been independently documented in both aquatic and terrestrial ecosystems (reviewed in Power 1992; Shurin et al. 2002). However, increasing evidence suggests that ecological interactions can cross these habitat and ecosystem boundaries, resulting in surprisingly strong direct and indirect effects among species in distinct environments (Wallace et al. 1997; Helfield and Naiman 2001; Baxter et al. 2004). For instance, over 80 % of all animal species have complex life cycles and undergo ontogenetic shifts in habitat affinity (Werner 1988), often alternating between the use of terrestrial and aquatic systems (Wilbur 1980; Werner and Gilliam 1984). Given this prevalence of complex life cycles, linkages across habitat and ecosystem boundaries are more common than previously recognized (McCoy et al. 2009; Wesner 2010).

Due to the propensity of many species to undergo aquatic-to-terrestrial life-stage progression, terrestrial systems may be particularly sensitive to local aquatic trophic dynamics. For instance, lentic fish can dramatically increase the fitness of nearby terrestrial plants via indirect effects of larval dragonfly consumption in the aquatic environment (Knight et al. 2005). By preying upon the larval life-stage, fish reduce the density of emerging adult dragonflies, which are important predators of terrestrial plant pollinators. Though the potential for reciprocal trophic effects between these ecosystems clearly exists, if and how terrestrial trophic interactions influence aquatic food webs is largely unexplored (see Carpenter et al. 2005; McCoy et al. 2009; Sato et al. 2011 for exceptions). Furthermore, a more thorough understanding of trophic linkages among habitats may reveal additional direct and indirect effects of habitat alteration.

Within terrestrial systems, invasive plants can have significant consequences for native plant community composition through competition and species replacement at a local scale (e.g., Vitousek et al. 1997; Levine et al. 2003). Native plant–pollinator interaction networks are also influenced by plant invasion (e.g., Aizen et al. 2008; Padron et al. 2009; Vila et al. 2009). However, little is known about how invasive plants might alter terrestrial and aquatic food webs simultaneously, despite the potential for strong consumer–resource interactions that could influence system dynamics via complex life cycle organisms. For example, the flowers of highly fertile invasive plants may subsidize insect populations by attracting large numbers of pollinators (Brown et al. 2002). Locally abundant insect pollinator prey may then attract a greater abundance of predatory adult dragonflies. This terrestrial, bottom-up effect resulting from increased prey resources could alter the food webs of nearby wetlands through increased dragonfly oviposition and recruitment of their aquatic larvae. Dragonfly larvae are voracious consumers of aquatic macroinvertebrates, and large changes in their abundances and foraging pressure could cascade through the aquatic community to influence lower trophic level taxa (Benke 1976, 1978; Batzer and Wissinger 1996).

**Fig. 1:**
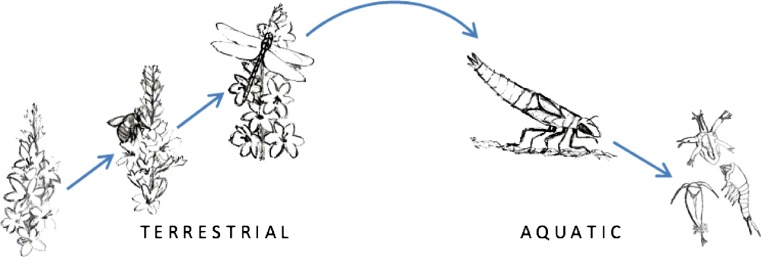
Conceptual diagram of the hypothesized trophic pathway in this study. We predicted that introductions of the prolifically blooming invasive plant purple loosestrife (*L. salicaria*) would attract insect pollinators and, subsequently, adult dragonflies, which prey upon the smaller insects. Through increased dragonfly oviposition and greater abundance of predaceous larval dragonflies in the wetland, these bottom-up terrestrial trophic interactions could then translate to top-down effects on the aquatic zooplankton community

Here, we investigate if and how purple loosestrife (*Lythrum salicaria*), a common, noxious invasive plant of wetland–terrestrial interfaces, affects terrestrial and aquatic trophic interactions in a manipulative pond study. Purple loosestrife can occur in high densities and dominate large expanses of wetland habitat, is highly fertile, and attracts large numbers of terrestrial, pollinating insects (Brown et al. 2002). Uninvaded wetlands typically have low floral densities relative to those invaded by purple loosestrife, through the displacement of native plants with no or low flowering (e.g., cattails, Mal et al. 1997; or native congener of *L. salicaria*, Brown et al. 2002). Thus, loosestrife is functionally different from the native plants it replaces, and this may have important community-level consequences. We chose purple loosestrife for this study to (1) investigate, in a basic ecological sense, the degree to which experimental differences in floral densities can cross the terrestrial–aquatic ecotone, and (2) provide a realistic ecological scenario in which such effects may occur. We hypothesized that the introduction of flowering purple loosestrife plants to artificial wetlands would stimulate a series of terrestrial trophic interactions in which bottom-up effects of loosestrife plants on secondary consumers would in turn generate top-down effects in the aquatic community, cascading down to influence the abundance and diversity of zooplankton communities (Fig. 1). Although Polis et al. (1997) argue that these types of community alterations could result from direct inputs of resources across an ecotone, in this system we expect loosestrife floral resource density to indirectly influence the aquatic community via changes in the frequency of dragonfly oviposition events and reproduction. By experimentally manipulating floral density, we addressed this gap in knowledge of trophic interactions at the terrestrial–aquatic boundary (e.g., Polis and Strong 1996; Baxter et al. 2005) by focusing on dragonflies, hypothesized key players in this system.

## Methods

Eight artificial wetlands were created at Washington University in the Tyson Research Center, St. Louis, MO, USA, in June of 2009. Each wetland was located in an old field habitat within a matrix of oak–hickory forest, and comprised a central vinyl stock tank (~1,300 L capacity) and four smaller surrounding pools (~100 L capacity) filled with well water on June 12. These artificial wetlands were each positioned within 80 m of existing water bodies across the landscape (mean distance to existing water: 45 m, range 27–77 m). The experimental wetlands were an average of 306 m from each other (range 163–516 m). Neither distance to nearest water nor distance to nearest experimental wetland influenced any of the response metrics (*P* > 0.15 in all cases). The central tanks were stocked on June 22 with approximately equivalent amounts of six species of aquatic macrophytes and three species of snails. We collected zooplankton and phytoplankton from local ponds using an 80 μm plankton net, and used aliquots of this mixture to inoculate each central tank. The remainder of the aquatic community, including amphibians, odonates, dipterans, coleopterans, and hemipterans, was permitted to assemble naturally via dispersal from the local species pool. We assume that these methods provide each mesocosm with the same species pool, but allow for some natural stochasticity in colonization and extinction dynamics. We therefore expect that initial community composition was variable among replicates, but do not expect that initial composition would be biased with respect to experimental floral treatment.

We placed 25 loosestrife plants in pots in each of the four small pools around each central pond on June 12. Plants were grown from cuttings derived from five parent plants. Plants from the five parent lineages were divided equally among the wetland replicates to account for potential genetic effects. The artificial wetlands were randomly assigned one of five loosestrife flowering treatments: 0 % flowers (*n* = 2), 25 % flowers (*n* = 1), 50 % flowers (*n* = 2), 75 % flowers (*n* = 1), or 100 % flowers (*n* = 2). Once the loosestrife plants began flowering (July 6), we maintained these treatments until September 1 by removing the appropriate number of flowers at each wetland by hand three times per week, relative to the number of open flowers at the 100 % flowers treatment wetlands. By September 1, the loosestrife plants were finished flowering. We did not clip flowering stalks outright because we wanted to maintain equivalent plant structure at all wetlands, which is known to affect odonate oviposition behavior (Remsburg and Turner 2009). Occasionally, we removed some flowers at one of the 100 % flowers wetlands in order for the two replicates to have an equal number of open flowers. The spatial separation of the experimental loosestrife pools from the central sampling pool prevented the input of plant litter and pollen to the central pool. Each wetland was surrounded by fencing to prevent loosestrife decimation by deer herbivory. Thus, the spatial scale of each artificial wetland represented initial stages of loosestrife invasion, with small patches easily accessible to deer. The fencing may have allowed species interactions to occur that would typically manifest at moderate stages of loosestrife invasion that are less accessible to deer.

We observed all small pools equally for visiting insects during peak activity (0900–1500) for eight weeks (July 6 to September 1, 2009). All insects were identified in the field to species or morphospecies [see Electronic supplementary material (ESM) 1], and their behavior was recorded in the form of time spent at the pool and number of flowers visited. Insects were also classified into one of five size categories, ranging from very small (e.g., some sweat bees and syrphid flies) to very large (e.g., carpenter bees and some butterflies). Each pool was observed for 10 min once per week during the eight weeks of the experiment, for a total of 320 observation minutes at each wetland. Pools were observed individually in order to accurately assess small floral insect visitors, and data were pooled for each wetland. Each wetland was also observed for dragonfly activity (10 min per week for eight weeks) in sunny, hot weather in random order. Dragonfly individuals were counted and identified to species (ESM 2) through binoculars, and their behaviors were recorded as the amount of time spent flying, perching, or ovipositing. Dragonfly abundance and behaviors (e.g., number of oviposition events, quantified as when an individual approached the tank and repeatedly dipped her abdomen in the water) were summed over the total observation time for each wetland.

At the end of the experiment in October 2009, each of the eight central tanks was thoroughly sampled for zooplankton and macroinvertebrates using methods similar to those employed by Chase et al. (2009). By sampling over ten weeks after the first oviposition events were observed (July 21), we provided ample time for developing dragonfly larvae to influence the aquatic community. We exhaustively sampled invertebrates within two 0.2 m^2^ chimney samplers and collected and preserved all individuals in ethanol for later identification in the laboratory. We sampled tank walls for surface-dwelling invertebrates with sweeps of a 25 cm wide rectangular net from the bottom to the top of each tank wall at four locations in each tank. We sampled zooplankton from each central tank with five collections using an integrated tube sampler to sample the entire water column. These five samples were combined (~15 L total) and were filtered through an 80 μm mesh zooplankton net into a 50 mL sample for later laboratory identification. All taxa were identified in the laboratory using standard keys and guides. When identification to species level was not possible, taxa were identified to morphospecies (ESM 3 and 4).

To determine the relationships between the loosestrife flower treatments and insect visitors, dragonfly abundance, dragonfly oviposition, and trophic levels of the aquatic community, we performed individual regressions using JMP (version 4.0.4). We used a partially replicated regression design to maximize our statistical power (Cottingham et al. 2005). There was a large amount of variation in the abundance of zooplankton among treatments (range: 87–1245 sampled zooplankton individuals per tank). To ensure that differences among treatments in zooplankton richness were not caused by differences in the number of individuals, we conducted an individual-based rarefaction analysis on the zooplankton data by sampling down to the lowest common abundance value. Because the number of loosestrife flowers naturally fluctuated over the course of the season, and we manipulated the number of flowers to maintain our intended floral treatments, we used the percent of loosestrife flowers (i.e., the treatment) in our analyses, because it is a clear and constant independent variable. A single wetland (25 % flowers treatment) was excluded from all aquatic community analyses due to accidental contamination by fish larvae during zooplankton inoculation.

We described and quantified differences in zooplankton communities among treatments using descriptive and inferential multivariate methods. We first ordinated the zooplankton communities using nonmetric multidimensional scaling (NMDS) based on Bray–Curtis similarity values. Because our experimental design had very low (or no) replication within treatment groups, we could not formally test for differences in zooplankton community structure among treatment groups. Instead, we tested for a correlation between difference in treatment group (percent of loosestrife flowers) and Bray–Curtis community similarity via a Mantel’s test. Finally, to identify key differences in zooplankton communities between treatment groups, we pooled all zooplankton into four major functional/taxonomic groups (rotifers, cladocerans, copepods, or ostracods) and then conducted a SIMPER analysis to identify the groups that contributed the greatest change in Bray–Curtis similarity values among treatments. The SIMPER analysis was restricted to the 100, 50, and 0 % flower treatment categories, each of which were replicated twice. Multivariate community analyses were conducted in PAST (Hammer et al. 2001) and R (R Development Core Team, version 2.13.1).

## Results

**Fig. 2:**
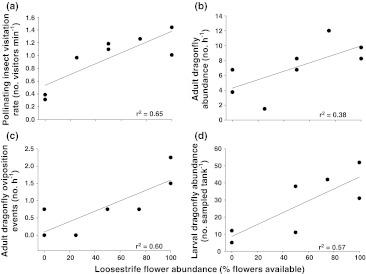
The abundances of **a** pollinating insects, **b** adult dragonflies, **c** dragonfly oviposition events, and **d** dragonfly larvae in wetlands increased with purple loosestrife (*L. salicaria*) flower abundance. Abundances were summed over equal sampling effort for each tank. The data points in each panel were fitted with a linear regression (*solid line*)

Insect visitation rates were higher in wetlands with greater numbers of loosestrife flowers available (Fig. 2a, *F*_1,6_ = 14.13, *P* = 0.0094, *r* ^2^ = 0.65, standardized regression coefficient = 0.84). There were no species-specific or size-specific trends in pollinator abundance or behavior across the floral density treatment (*P* > 0.20 in all cases). In addition, we found evidence of a positive relationship between loosestrife flower treatment and number of adult dragonflies that visited the experimental wetlands (Fig. 2b, *F*_1,6_ = 5.37, *P* = 0.059, *r* ^2^ = 0.38, standardized regression coefficient = 0.69) and number of dragonfly oviposition events (Fig. 2c, *F*_1,6_ = 11.37, *P* = 0.015, *r* ^2^ = 0.60, standardized regression coefficient = 0.81).

**Fig. 3:**
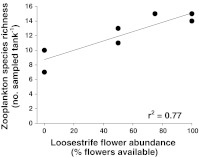
Zooplankton species richness increased with loosestrife flower abundance. Zooplankton richness was based on counts from 15 L of water from each tank. The data points were fitted with a linear regression (*solid line*)

At the end of the experiment, the abundance of dragonfly larvae in our aquatic samples was positively related to loosestrife flower treatment (Fig. 2d, *F*_1,5_ = 8.87, *P* = 0.031, *r* ^2^ = 0.57, standardized regression coefficient = 0.80). At the lower aquatic trophic level, raw (Fig. 3, *F*_1,5_ = 21.25, *P* = 0.0058, *r* ^2^ = 0.77, standardized regression coefficient = 0.90) and rarefied (*F*_1,5_ = 20.87, *P* = 0.0060, *r* ^2^ = 0.81, standardized regression coefficient = 0.90) zooplankton species richness was highly positively associated with loosestrife flower treatment. Neither larval dragonfly species richness (*F*_1,5_ = 1.39, *P* = 0.29) nor zooplankton abundance (*F*_1,5_ = 2.392, *P* = 0.18) were significantly associated with loosestrife flower treatment. Analysis of all other macroinvertebrates present in the aquatic community showed no significant relationships between flower treatment and either macroinvertebrate abundance (*F*_1,5_ = 0.32, *P* = 0.60) or richness (*F*_1,5_ = 0.077, *P* = 0.79). Likewise, when the predatory subset of macroinvertebrates was considered, we found no significant relationships between their abundance or richness and flower treatment (*F*_1,5_ = 0.077, *P* = 0.42 and *F*_1,5_ = 0.008, *P* = 0.93, respectively).

Zooplankton communities in similar flower treatment categories were more similar to one another than those in widely differing treatments (ESM Fig. 1, observed correlation value = −0.28, mean simulated value ± SD = 0.0045 ± 0.2060, *P* = 0.0731). Differences in zooplankton communities among treatments were primarily driven by differences in the abundance of rotifers, with greater rotifer abundance in 0 % flower treatments compared to 50 and 100 % flower treatments (Table 1). Copepods and cladocerans were also more abundant in the 0 % flower treatment than in the 100 % flower treatment.

**Table 1:** Results of a SIMPER analysis on differences in zooplankton functional/taxonomic groups among treatments. Analysis is based on Bray–Curtis distances among zooplankton communities

Taxonomic/functional group	Contribution	Cumulative %	Mean abundance within treatment		
			100 %	50 %	0 %
Rotifers	38.61	71.67	79	24.5	601
Copepods	9.45	89.22	38.5	75	73
Cladocerans	5.22	98.91	5.5	11	18.5
Ostracods	0.59	100	2.5	1.5	0

## Discussion

Organisms, nutrients, and energy are increasingly observed to cross traditional ecosystem boundaries (Power and Rainey 2000; Baxter et al. 2005). Here, we showed the capacity of the invasive wetland plant purple loosestrife (*L. salicaria*) to spark a series of terrestrial-to-aquatic trophic interactions, enhancing pollinator and predatory adult dragonfly local abundance, increasing dragonfly oviposition events in our experimental wetlands, increasing predatory larval dragonfly abundance, and altering zooplankton species richness as well as community structure in the aquatic community.

We documented a significant positive relationship between the density of purple loosestrife flowers and the visitation of flying insects, including many potential pollinators. Previous research has demonstrated that purple loosestrife is highly attractive to pollinating insects, and usurps pollinators from native congeners (Brown et al. 2002). Our results further suggest that, by attracting relatively high levels of pollinating insects where there might otherwise be little insect activity (Fig. 2a), purple loosestrife flowers potentially created a new resource base for novel trophic interactions. For instance, we documented a concomitant increase in adult dragonfly abundance with increasing loosestrife flower levels. Adult dragonflies are predators of many small flying insects (Corbet 1999; Knight et al. 2005). These results suggest that adult dragonflies respond with increased abundance and activity to the presence of flying insect prey, to the visual cue of the flowers themselves, or to both. The nature of our experimental design does not allow us to separate and assess the relative importance of these potential effects. We are confident, however, that by removing only flowers and not plant stems we were able to rule out the known effects of plant structure on dragonfly activity and abundance (Remsburg and Turner 2009).

Our results also indicate that the adult dragonfly abundances associated with high-flower treatments resulted in a higher frequency of dragonfly oviposition events and a subsequently higher abundance of dragonfly larvae in the experimental ponds as compared to low-flower treatments. This is a key link between the terrestrial and aquatic food webs, with a bottom-up effect of increased adult dragonflies resulting in higher densities of aquatic predators (dragonfly larvae) and in the potential for a top-down aquatic trophic cascade. Further experiments are required to mechanistically confirm each step of this cascade beyond what we observed from floral manipulation alone.

Finally, we document a strong positive relationship between flower treatment level and zooplankton species richness. Although we hypothesized that flower treatment level would influence lower trophic levels in the aquatic environment, we did not predict a priori that this effect would manifest itself as elevated zooplankton richness in high-flower wetlands, with no overall effect on zooplankton abundance. Although a bottom-up effect of increased inputs of pollen or detritus from purple loosestrife is one possible explanation for this result, we consider it to be unlikely for three reasons. First, purple loosestrife is primarily insect, not wind, pollinated. Second, the loosestrife plants were housed in separate aquatic mesocosms and could therefore not deposit detritus in the central mesocosms that were the focus of our aquatic sampling. Third, rarefied zooplankton richness also increased with loosestrife flower treatment, which suggests that differences in richness among the treatments were not driven by differences in abundance; this contrasts with a bottom-up (more individuals) effect on zooplankton.

A second, and more likely, explanation is that higher zooplankton richness in high-flower treatment ponds was mediated by higher densities of dragonfly larvae, possibly via a keystone effect of predation by dragonfly larvae. We have evidence of large shifts in abundance patterns of zooplankton taxonomic categories (Table 1), which in combination with the positive relationship between flower treatment and zooplankton richness, is consistent with a keystone effect. We also found evidence of a shift in zooplankton community composition associated with flower treatment level, such that similar flower treatments (and similar abundance of larval dragonflies) resulted in more similar zooplankton communities (Mantel’s test and ESM Fig. 1). This general pattern suggests that in this experiment, predation by dragonfly larvae was selective and altered the structure of the zooplankton communities, potentially toward more similar, species-rich communities. This result is further supported by evidence that this change in aquatic community structure was primarily driven by a reduction in the abundance of rotifers. Although significant effects of dragonfly predation on zooplankton assemblages have been documented relatively rarely and are variable among studies (e.g., Hampton and Gilbert 2001; Burks et al. 2001; Magnusson and Williams 2009), dragonfly larvae are known to prey on rotifers to varying degrees (Hampton and Gilbert 2001; Walsh et al. 2006). This effect may have been particularly strong in our study, in which a large proportion of dragonfly larvae were of small size.

Other mechanisms may also contribute to the positive relationship between floral treatment and zooplankton richness, including increased zooplankton colonization opportunities associated with increased dragonfly oviposition events (Havel and Shurin 2004). Intraguild predation, seen as the suppression of other macroinvertebrate predators by high densities of dragonfly larvae, is also a possible mechanism. However, we did not detect any associations between flower treatments and the abundance or richness of non-odonate macroinvertebrate predators in this study. Finally, we cannot rule out other indirect effects of larval odonate abundance on zooplankton richness. The majority of the dragonfly larvae present in our experimental ponds were small in size, supporting effects on zooplankton and not other taxa.

Overall, our results demonstrate that trophic effects generated by an emergent wetland invasive flowering plant are propagated into the aquatic system by a common group of insects with a complex lifecycle: dragonflies. Our results were surprising in that the effects of purple loosestrife were consistently strong and operated across four distinct levels of trophic interactions. Our study was short-term, highly manipulative, and focused on the assembly phase of the aquatic food web, when dragonflies (for example) may be recruitment limited. Whether effects such as those observed here also occur in natural habitats in mature wetlands is unknown. However, the importance of assembly history in determining the long-term trajectory of ecological communities suggests that effects similar to those we document here could potentially have long-lasting consequences for the structure of aquatic communities. Documenting the strength of longer-term reciprocal effects (e.g., Baxter et al. 2005; Massol et al. 2011) of loosestrife across the aquatic–terrestrial ecotone will be an important goal for future work in this and similar systems.

Invasive plants have well-documented effects on terrestrial communities. Purple loosestrife can directly influence native plant communities through competition for resources by reducing the colonization success of native species and outcompeting rare species (Hovick et al. 2011). Purple loosestrife can also indirectly reduce native plant fitness by usurping pollinators (Brown et al. 2002) or altering abiotic conditions that influence pollinator visitation, such as the light environment (McKinney and Goodell 2010). Invasive wetland plants like purple loosestrife also have the potential to link and disrupt native terrestrial and aquatic ecosystems (Naiman and Decamps 1997) via allochthonous resource inputs and alterations of aquatic communities (e.g., Schulze and Walker 1997; Bailey et al. 2001; Going and Dudley 2007), leading to an overall alteration of community structure and ecosystem functioning (Hladyz et al. 2011). However, less studied are the multi-trophic interactions that may be propagated across ecosystem boundaries through behavioral responses to resource availability and ontogenetic habitat shifts. Our results suggest that purple loosestrife, as a plant that produces much more floral resources than the native plants that it replaces, has the capacity to influence the attraction of predatory adult dragonflies that link terrestrial and aquatic trophic interactions. Further experimentation incorporating a native floral community as an additional control will help to quantify the relative magnitude of effects on aquatic communities. Given that purple loosestrife can dominate large areas of wetlands, its invasion might have important implications for species interactions and trophic structure over large scales. Larger-scale studies will help elucidate the landscape implications of loosestrife invasion, particularly in the context of population-level pollinator and dragonfly foraging behavior. The effects of invasive species may propagate further and through more cryptic pathways than previously appreciated. By broadening our views of ecosystems and collaborating across traditional disciplines, terrestrial and aquatic ecologists together may better understand the intricacies of trophic interactions.

## Electronic supplementary material

Below is the link to the electronic supplementary material.

Supplementary material 1 (DOCX 64 kb)
