# Supplementary material for: Effects of an invasive plant transcend ecosystem boundaries through a dragonfly-mediated trophic pathway
Source: Oecologia. 2012 May 24;170(4):1045–52. doi: 10.1007/s00442-012-2357-1 (PMC3496476; doi:10.1007/s00442-012-2357-1)
Supplement: Supplementary file 1 — Supplementary material 1 (DOCX 64 kb) [file 442_2012_2357_MOESM1_ESM.docx]

ESM 1. List of floral insect visitors

| *Aedes sp.* |
| --- |
| *Apis mellifera* |
| *Archilochus colubris* |
| *Archytas sp.* |
| *Asclera ruficollis* |
| *Augochlora pura* |
| *Augochlorella aurata* |
| *Battus philenor* |
| *Bombus impatiens* |
| *Calliphoridae sp. 1* |
| *Calliphoridae sp. 2* |
| *Calliphoridae sp. 3* |
| *Ceratina sp.* |
| *Cerceris sp.* |
| *Chauliognathus pennsylvanicus* |
| *Cisseps fulvicollis* |
| *Colias sp.* |
| *Cupido comyntas* |
| *Epargyreus clarus* |
| *Eristalinus aeneus* |
| *Eristalis transversa* |
| *Eupeodes sp.* |
| *Hemiptera sp. 1* |
| *Hylaeus sp.* |
| *Lasioglossum sp.* |
| *Lycaenidae sp. 1* |
| *Megachilid sp.* |
| *Melanoplus sp.* |
| *Nomada sp.* |
| *Pieris sp. 1* |
| *Poanes hobomok* |
| *Polistes dominula* |
| *Polistes fuscatus* |
| *Polistes metricus* |
| *Sarcophagidae sp.* |
| *Simulium sp.* |
| *Speyeria cybele* |
| *Sphaerophoria sp.* |
| *Stenodynerus sp.* |
| *Syrphus sp.* |
| *Tabanus sp.* |
| *Vespula sp.* |
| *Xylocopa virginica* |

ESM 2a. List of adult dragonfly species

*Anax junius*

*Erythemis simplicicollis*

*Libellula luctuosa*

*Libellula saturata*

*Libellula pulchella*

*Pachydiplax longipennis*

*Tramea lacerata*

ESM 2b. List of larval dragonfly species

*Anax junius*

*Erythemis simplicicollis*

*Libellula pulchella*

*Libellula saturata*

*Libellula sp.*

*Pachydiplax longipennis*

*Pantala flavescens*

ESM 3. List of zooplankton species present (“1”) and absent (“0”) in each treatment.

|  | Treatment | | | |
| --- | --- | --- | --- | --- |
| Species | 0% | 50% | 75% | 100% |
| *Asplanchna priodonta* | 1 | 1 | 1 | 1 |
| *Bosmina longirostris* | 0 | 0 | 1 | 0 |
| *Brachionus angularis* | 0 | 0 | 1 | 1 |
| *Brachionus quadridentata* | 1 | 0 | 0 | 0 |
| *Ceriodaphnia lacustris* | 1 | 1 | 1 | 1 |
| *Chydorus sphaericus* | 1 | 1 | 1 | 1 |
| *Diaphanosoma birgei* | 1 | 1 | 1 | 1 |
| *Keratella cochlearis* | 1 | 0 | 1 | 1 |
| *Keratella valga* | 1 | 1 | 1 | 1 |
| *Lecane crepida* | 1 | 1 | 1 | 1 |
| *Lecane stokesi* | 0 | 1 | 1 | 0 |
| *Lecane tudicola* | 1 | 1 | 0 | 0 |
| *Lecane ungulata* | 1 | 0 | 1 | 1 |
| *Mesocyclops edax* | 1 | 1 | 0 | 0 |
| *Microcyclops rubellus* | 1 | 1 | 1 | 1 |
| *Monostyla angularis* | 1 | 0 | 0 | 0 |
| *Monostyla bulla* | 1 | 1 | 1 | 1 |
| Nauplius | 1 | 1 | 1 | 1 |
| *Notholca foliea* | 0 | 0 | 1 | 0 |
| Ostrocod | 1 | 1 | 1 | 0 |
| *Platyias patulus* | 0 | 0 | 1 | 1 |
| Rotifer F | 1 | 1 | 1 | 1 |
| Rotifer A | 1 | 1 | 0 | 1 |
| Rotifer B | 0 | 0 | 1 | 0 |
| Rotifer G | 0 | 0 | 0 | 1 |
| Rotifer H | 0 | 0 | 0 | 1 |
| Rotifer I | 0 | 0 | 1 | 0 |
| *Skistodiaptomus sp.* | 1 | 0 | 0 | 0 |
| Trichocerca A | 0 | 0 | 1 | 0 |
| Trichocerca B | 1 | 1 | 0 | 0 |

ESM 4. List of other macroinvertebrate species

| *Acilius fraternus* |
| --- |
| *Anopheles sp.* |
| *Belostoma sp.* |
| *Berosus sp. 1* |
| *Callibaetis sp.* |
| *Chaoborus sp.* |
| *Chironomid sp.* |
| *Copelatus sp.* |
| *Gyraulus parbus* |
| *Helisoma trivolis* |
| *Hesperocorixa sp.* |
| *Hydroporus sp. 2* |
| *Hygrotus sp. 2* |
| *Ischnura sp.* |
| *Laccophilus maculosus* |
| *Notonecta undulata* |
| *Notonecta irrorata* |
| *Notonectid Nymph* |
| *Peltodytes litoralis* |
| *Petlodytes sp. larvae* |
| *Physa gyrina* |
| *Tropisternus blatchleyi* |
| *Tropisternus lateralis* |
| *Tropisternus collaris* |
| *Sigara sp.* |

ESM Figure 1. Nonmetric multidimensional scaling of zooplankton communities across loosestrife flower treatment groups. Distances are based on Bray-Curtis similarity values. Circles = 0% flower treatment; hollow squares = 50% flower treatment; filled square = 75% flower treatment; crosses = 100% flower treatment. Stress = 0.049.

-0.32

-0.16

0

0.16

0.32

0.48

0.64

0.8

Coordinate 1

-0.25

-0.2

-0.15

-0.1
